# Probing hepatic metabolism of [2-^13^C]dihydroxyacetone in vivo with ^1^H-decoupled hyperpolarized ^13^C-MR

**DOI:** 10.1007/s10334-020-00884-y

**Published:** 2020-09-10

**Authors:** Irene Marco-Rius, Alan J. Wright, De-en Hu, Dragana Savic, Jack J. Miller, Kerstin N. Timm, Damian Tyler, Kevin M. Brindle, Arnaud Comment

**Affiliations:** 1 Cancer Research UK Cambridge Institute, University of Cambridge, Cambridge, UK; 2 Department of Physiology, Anatomy and Genetics, University of Oxford, Oxford, UK; 3 Clarendon Laboratory, Department of Physics, University of Oxford, Oxford, UK; 4 Oxford Centre for Clinical Magnetic Resonance Research, Radcliffe Division of Medicine, University of Oxford, Oxford, UK; 5 General Electric Healthcare, Chalfont St Giles, UK; 6 Present Address: Institute for Bioengineering of Catalonia, Barcelona, Spain

**Keywords:** Carbon-13 magnetic resonance spectroscopy, Gluconeogenesis, Glycolysis, Liver, Metabolism, Dynamic Nuclear Polarisation, Hyperpolarisation

## Abstract

**Objectives:**

To enhance detection of the products of hyperpolarized [2-^13^C]dihydroxyacetone metabolism for assessment of three metabolic pathways in the liver in vivo. Hyperpolarized [2-^13^C]DHAc emerged as a promising substrate to follow gluconeogenesis, glycolysis and the glycerol pathways. However, the use of [2-^13^C]DHAc in vivo has not taken off because (i) the chemical shift range of [2-^13^C]DHAc and its metabolic products span over 144 ppm, and (ii) ^1^H decoupling is required to increase spectral resolution and sensitivity. While these issues are trivial for high-field vertical-bore NMR spectrometers, horizontal-bore small-animal MR scanners are seldom equipped for such experiments.

**Methods:**

Real-time hepatic metabolism of three fed mice was probed by ^1^H-decoupled ^13^C-MR following injection of hyperpolarized [2-^13^C]DHAc. The spectra of [2-^13^C]DHAc and its metabolic products were acquired in a 7 T small-animal MR scanner using three purpose-designed spectral-spatial radiofrequency pulses that excited a spatial bandwidth of 8 mm with varying spectral bandwidths and central frequencies (chemical shifts).

**Results:**

The metabolic products detected in vivo include glycerol 3-phosphate, glycerol, phosphoenolpyruvate, lactate, alanine, glyceraldehyde 3-phosphate and glucose 6-phosphate. The metabolite-to-substrate ratios were comparable to those reported previously in perfused liver.

**Discussion:**

Three metabolic pathways can be probed simultaneously in the mouse liver in vivo, in real time,  using hyperpolarized DHAc.

**Electronic supplementary material:**

The online version of this article (10.1007/s10334-020-00884-y) contains supplementary material, which is available to authorized users.

## Introduction

The human liver performs a broad range of tasks that affect the function of the whole body, from maintaining plasma glucose homeostasis to filtering toxic chemicals from food and drinks [1]. In most cases, the liver can regenerate after an insult and recover functionality. However, recurrent damage to the liver may result in permanent loss of liver mass, with potentially fatal consequences.

Magnetic resonance (MR) could play an important role in the identification of biomarkers to assess metabolic function of the liver in situ and non-invasively. For example, multiparametric ^1^H-MR has already proven to be a valuable tool to stratify NAFLD patients [2, 3], and MRI-estimated proton density fat fraction and MR elastography allow quantification of hepatic steatosis and fibrosis, respectively [4]. In ^13^C-MR, resonances are widely dispersed over a chemical shift range of ~ 200 ppm, and multiple metabolites can easily be distinguished. However, the clinical value of ^13^C-MR has been limited by its intrinsically low sensitivity compared to ^1^H-MR due to (1) its low natural abundance, (2) lower nuclear polarization, a consequence of the four times lower gyromagnetic ratio of the carbon-13 nucleus, and, where molecularly relevant, (3) splitting of the ^13^C resonances due to spin–spin coupling with protons. The first two limitations may be overcome by injecting hyperpolarized (HP) ^13^C-labeled molecules, which transiently boost the ^13^C-MR signal. The third limitation can be resolved with ^1^H decoupling during ^13^C signal reception.

HP-MR by dissolution Dynamic Nuclear Polarization (DNP) is an emerging technique that increases the sensitivity of the MR signal by 50,000-fold. A single DNP shot (~ seconds) can provide a signal-to-noise ratio (SNR) that is not accessible by any conventional MR acquisition. Consequently, this hyper-intense signal can be used to measure rapid metabolic processes such as enzymatic reactions and transient metabolic reaction intermediates in vivo, in situ and non-invasively with high temporal resolution. DNP-MR can provide information on the mechanistic and biochemical changes that occur in the diseased organ [5]. Multiple HP ^13^C-labeled substrates have provided insights into several metabolic pathways, including glycolysis, the pentose-phosphate pathway and cellular redox state [6, 7]. And strategies are being explored to increase organ selectivity by suppressing the signal arising from specific cell types [8]. HP [1-^13^C]pyruvate has been used extensively in preclinical studies of cancer, cardiovascular diseases and diabetes, and has already been translated into a clinical setting [7, 9]. Another HP probe, [2-^13^C]dihydroxyacetone (DHAc), has recently been shown to report on glucose metabolism in the liver [10, 11] and could potentially be used to investigate the dysregulation of hepatic gluconeogenesis (GNG) and glycolysis in metabolic diseases such as NAFLD [12]. HP [U-^2^H,^13^C]glucose has also been used successfully for in vivo metabolic studies [13, 14]. However, with current experimental setups, the loss of ^13^C polarization during transport (3–15 s) and substrate delivery to the organ of interest (~ 10 s) is far less for [2-^13^C]DHAc (*T*_1_ ~ 40 s) than for [U-^2^H,^13^C]glucose (*T*_1_ < 15 s), making DHAc a superior probe for sampling GNG and glycolysis in vivo.

**Fig. 1:**
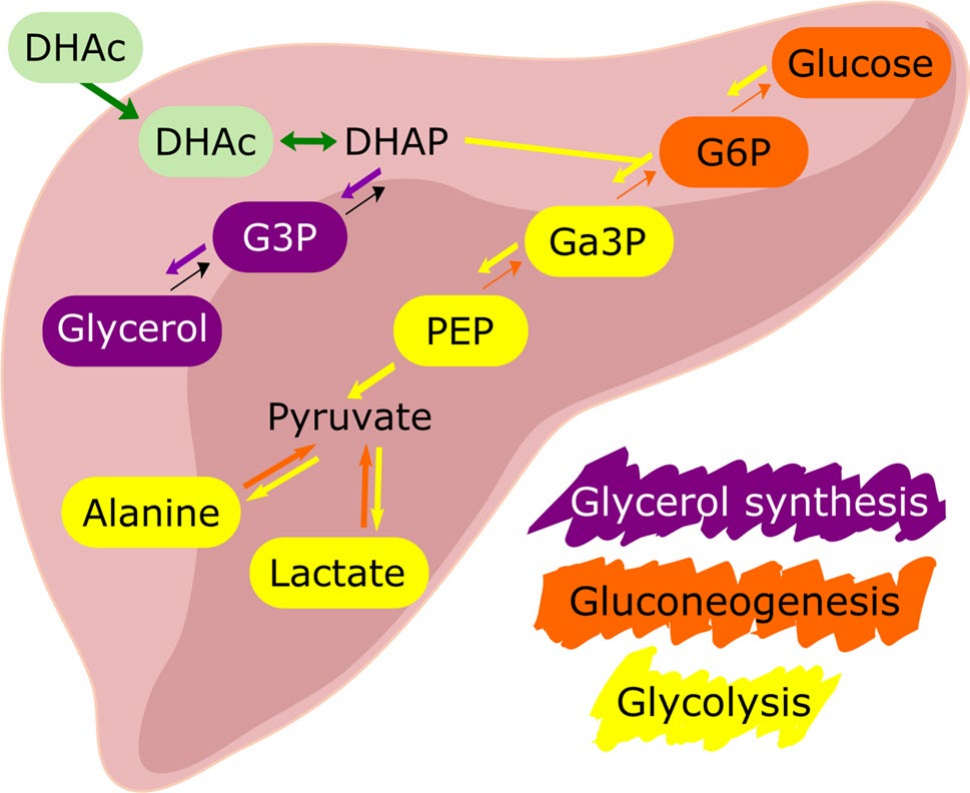
Metabolism of dihydroxyacetone in the liver. Metabolites highlighted in either purple (glycerol synthesis) or yellow (gluconeogenesis/glycolysis) were observed in the present ^13^C-MR study. *DHAc* dihydroxyacetone, *DHAP* dihydroxyacetone phosphate, *G3P* glycerol-3-phosphate, *G6P* glucose-6-phosphate, *Ga3P* glyceraldehyde 3-phosphate, *PEP* phosphoenolpyruvate

Upon injection, DHAc is rapidly converted into dihydroxyacetone phosphate (DHAP) and enters the glycolytic, gluconeogenic and glycerol synthesis pathways. This imaging agent probes the upper half of GNG as DHAP is converted to fructose-1,6-bisphosphate and on to glucose, potentially via glyceraldehyde-3-phosphate (Ga3P). Similarly, DHAc probes the lower half of glycolysis as DHAP via Ga3P is converted to glycerate-1,3-bisphosphate (Fig. 1). The oral and transdermal administration of DHAc has an exceptional safety profile, having been given as an oral metabolic challenge nearly a century ago [15], and currently being used as a tanning product [16]. In addition, DHAc has recently been hyperpolarized using photogenerated non-persistent radicals, which results in a solution of HP DHAc free of radicals without the need for filtration prior to injection into a patient [7]. Although DHAc has clinical translational potential, any eventual clinical translation would be dependent on phase-1 trials to ensure that the agent was safe for patients with compromised liver function. In perfused liver, ^1^H-decoupled ^13^C-MR spectra showed that [2-^13^C]DHAc metabolism could report on GNG, glycolysis and glycerol synthesis pathways [10]. Such detail of metabolism has not previously been observed in vivo. An initial study using a 3 T clinical scanner in which ^1^H-decoupling was applied during non-selective, broadband pulse and acquire ^13^C-MR spectroscopy showed that ^1^H-decoupling increased the signal-to-noise ratio (SNR) of glycerol-3-phosphate (G3P) by 71%, albeit G3P was the only metabolite that could be detected apart from the injected HP [2-^13^C]DHAc and its hydrate [17]. The challenges inherent to in vivo ^13^C-MR experiments with HP [2-^13^C]DHAc are due to the large chemical shift range covered by the substrate and its products [18] (~ 144 ppm or ~ 10.5 kHz at 7 T). These challenges include (i) requiring a spatial localisation by excitation to restrict signals to the tissue of interest and, at the same time, (ii) requiring a reduced excitation of the spectral bandwidth to avoid large chemical shift artefact that would be induced from a single 10.5 kHz bandwidth; (iii) excessive excitation of the HP sample magnetization, which shortens the time window for real-time metabolic fate observation. To mitigate these undesirable effects, algorithmically generated RF pulses may be designed and used to excite MR signals with spectral and spatial (SPSP) control [11, 18]. A single SPSP RF pulse probing the entire spectral range covered by DHAc and its downstream products has been successfully implemented at 3 T (~ 4.6 kHz spectral bandwidth) [11, 18]. However, spectral constraints on pulse design favor SPSP RF control at narrow spectral bandwidths, and at higher magnetic fields, SPSP selectivity is better controlled by multiple SPSP RF pulses applied sequentially [19].

Here, we present a protocol that involves ^1^H-decoupled ^13^C-MR acquisitions using SPSP RF pulses for the detection of HP [2-^13^C]DHAc in the mouse liver in vivo at 7 T (~ 10.5 kHz spectral bandwidth) and the same metabolic products that were observed in the perfused mouse liver at 9.4 T [10] with comparable spectral resolution (linewidth < 20 Hz in vivo). The study was designed to enhance detection of the metabolic products of HP [2-^13^C]DHAc and to allow for simultaneous assessment of three metabolic pathways in the liver in vivo.

## Experimental

### Spectral–spatial (SPSP) RF pulse design

**Fig. 2:**
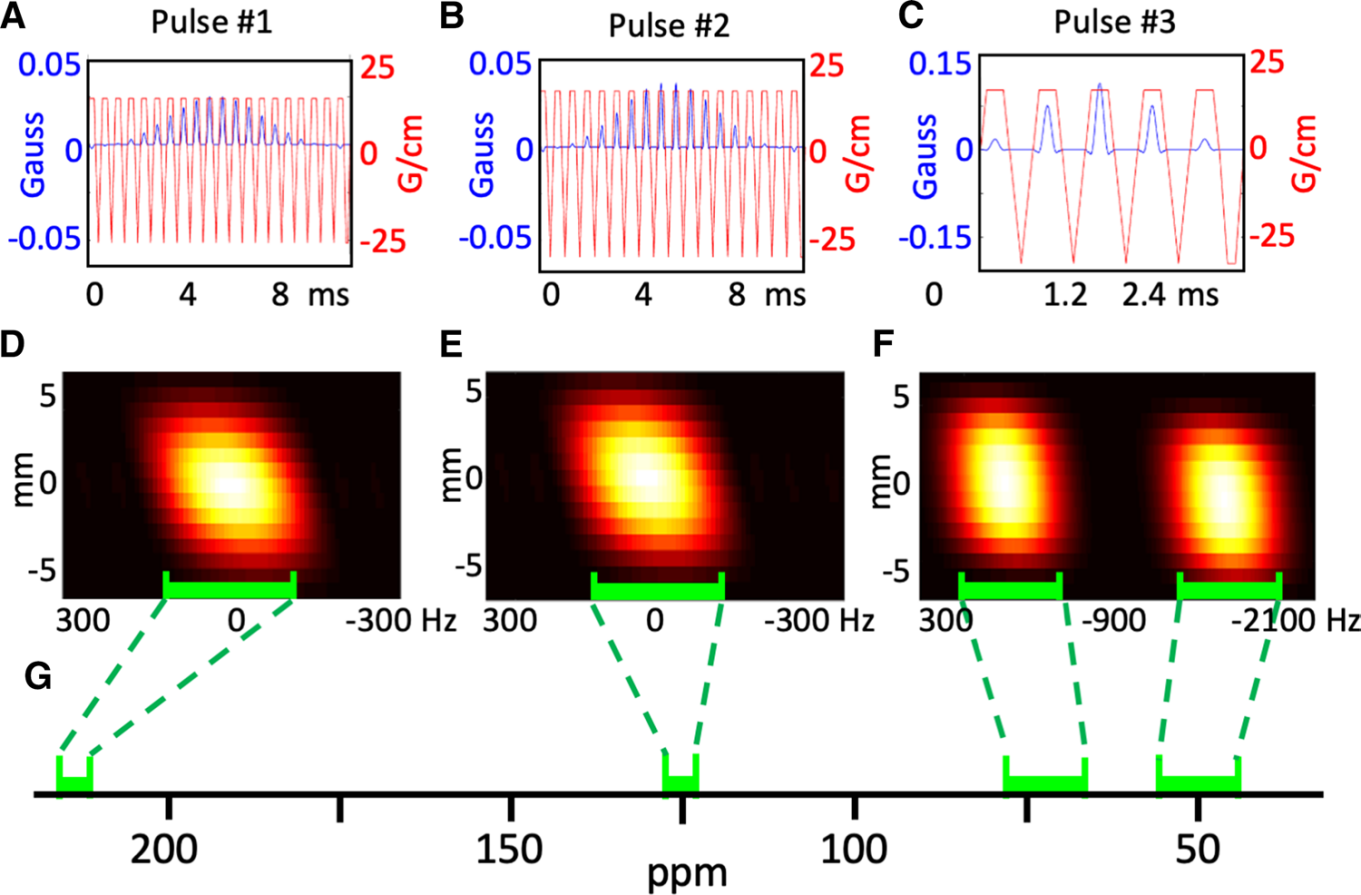
Radiofrequency (blue; G) and magnetic field gradient (red; G/cm) profiles of the spectral-spatial RF pulses used to excite **a** dihydroxyacetone (DHAc), **b** phosphoenolpyruvate (PEP) and **c** other metabolites of interest. Simulations of their frequency and spatial profiles, shown as transverse magnetization, for the three pulses are shown in: (**d**) DHAc, (**e**) PEP and (**f**) the other metabolites of interest. **g** A chemical shift axis showing the regions of the spectrum that are excited by the spectral-spatial pulses

Three SPSP RF pulses were designed using a MATLAB® software package available online (https://github.com/agentmess/Spectral-Spatial-RF-Pulse-Design) [20, 21] to excite a 8 mm thick slab with varying central frequencies and spectral bandwidths (Fig. 2): ‘pulse #1’ was centered on the [2-^13^C]DHAc resonance (214 ppm, 250 Hz spectral passband, 1908 Hz spectral stop-band, 6757 Hz spatial bandwidth, 10.44 ms), ‘pulse #2’ on the phosphoenolpyruvate (PEP) resonance (150.5 ppm, 250 Hz spectral passband, 1724 Hz spectral stop-band, 7813 Hz spatial bandwidth, 10.18 ms), and ‘pulse #3’ was set to excite the other metabolites of interest (72 ppm and 50 ppm, 780 Hz spectral passband, 1634 Hz spectral stop-band, 8929 Hz spatial bandwidth, 2.98 ms). The RF pulses used a flyback design and were optimized to excite the frequencies of the resonances of interest while avoiding excitation of the other resonances by the periodic excitation bands. An extra gradient lobe was used to refocus spins across the spatial dimension and a maximum slew rate of 2 × 10^5^ Gauss/cm/s (2000 T/m/s) and raster time of 4 μs were included as a constraint on the pulse design.

### Hyperpolarization of [2-^13^C]DHAc

Hyperpolarized samples of [2-^13^C]DHAc were prepared as described previously [18]^.^ Briefly, a stock solution of 8 M [2-^13^C]DHAc (Sigma-Aldrich, Haverhill, UK) and 21 mM trityl radical OX063 (GE Healthcare, Amersham, Buckinghamshire, UK) in 2:1 water:dimethyl sulfoxide (v/v) was sonicated for 5 min at 40 ºC. On the day of the experiments, 1.2 mM gadoteric acid (Dotarem, Guerbet, Roissy, France) was added to 40 μl of the stock solution. The sample was then hyperpolarized by dynamic nuclear polarization (DNP) at 3.35 T and 1.25 K (HyperSense, Oxford Instruments, Abingdon, UK) for approximately 80 min (build up time constant: 1360 ± 3 s; polarization level: P ~ 16% at 16 s after dissolution) and rapidly dissolved in 6 mL of a superheated solution of phosphate-buffered saline (neutral pH after dissolution).

### Hyperpolarized MR studies in vivo

All animal studies were carried out under explicit project (1840186)  and personal licenses approved by the UK Home Office following independent ethical review by the Cancer Research UK, Cambridge Institute Animal Welfare and Ethical Review Body. Three female C57B6 mice (weight = 20–25 g) were used in these experiments. To avoid changes due to circadian rhythm, the three animals were allowed to feed (ad libitum water and food access) during scotophase (nocturnal feed represents 75% of the total food intake [22]) and all MR acquisitions were performed during the first hours of the photophase, while the animals were still in a fed state (between 8 am and 1 pm). Each mouse was anaesthetized with 2% isoflurane/oxygen mixture and a tail-vein cannula inserted for HP [2-^13^C]DHAc administration. A 10-mm diameter ^13^C receive surface coil (Rapid Biomedical GMBH, Rimpar, Germany) was placed over the liver of the mouse, which was in a prone position, prior to moving the animal into a 7 T small-animal MR scanner (Agilent, Palo Alto, CA) equipped with 400 mT/m, 3000 T/m/s gradient coils and a broadband RF amplifier, with temperature and breathing rate monitoring.

**Fig. 3:**
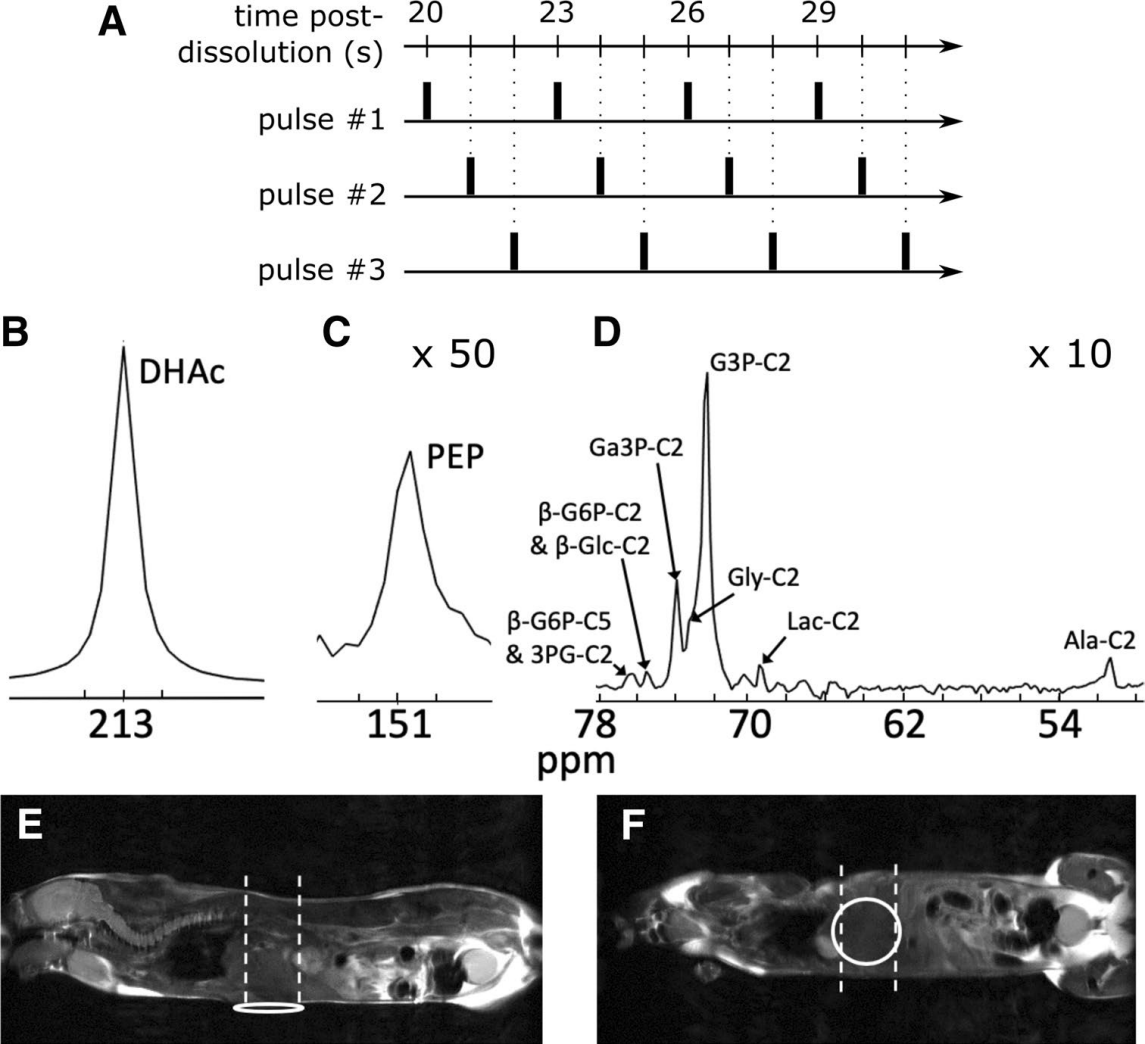
Acquisition scheme and spectra acquired from the liver of a mouse infused with hyperpolarized [2-13C]dihydroxyacetone (DHAc). **a** Three spectral regions were acquired in an interleaved fashion. **b** Spectrum showing DHAc resonance at 212.9 ppm (acquired using pulse #1). **c** Spectrum (×10 vertical scale relative to (**b**)) showing the phosphoenolpyruvate (PEP) resonance (acquired using pulse #2). **d** Spectrum (×10 vertical scale relative to (**b**)) showing resonances from C5-β-glucose-6-phosphate (G6P-C5), C2-3-phosphoglycerate (3PG-C2), C2-β-glucose (Glc-C2), C2-β-glucose-6-phosphate (G6P-C2), C2- glyceraldehyde 3-phosphate (Ga3P), C2-glyceraldehyde-3-phosphate (Ga3P-C2), C2 -glycerol (Gly-C2), C2-glycerol-3-phosphate (G3P-C2), C2-lactate (Lac-C2) and C2-alanine (Ala-C2) (acquired using pulse #3). **e**, **f** T2-weighted images of the mouse (84 × 42 mm 512 × 256 data points, fast-spin-echo; effective echo time 25 ms; repetition time 1.5 s). The location and orientation of the 10-mm receive coil is represented by the bold white line and the 8- mm- thick acquisition slice is indicated by the dotted lines

RF pulses were applied using a 42 mm diameter volume ^1^H transmit/receive and ^13^C transmit coil (Rapid Biomedical GMBH, Rimpar, Germany), and the ^13^C signal recorded through the 10-mm ^13^C surface coil. Sagittal, coronal and axial T_2_-weighted ^1^H MR images were acquired for anatomical reference and to confirm the positioning of the surface coil. A slice selective pulse acquire ^1^H-sequence was used to manually shim the 8 mm spatial slice of the ^13^C acquisition. Localized, ^1^H-decoupled, dynamic ^13^C-MR spectra were acquired from 20 s after the beginning of the injection of HP [2-^13^C]DHAc (400 μl of 54 mM DHAc solution injected over 3 s, ~ 10 s after dissolution), using a series of three consecutive SPSP RF pulse-acquisitions (Fig. 3a). Inverse-gated ^1^H-decoupling was applied only during signal acquisition (Waltz 16, 0.235 Gauss). Acquisition parameters included: repetition time for dynamic acquisition = 1 s; receiver bandwidth = 10 kHz; number of points = 2048; # scans at each acquisition frequency = 9; nominal flip angle pulse #1 = 15º at the DHAc resonance, nominal flip angle pulse #2 = 90º, nominal flip angle pulse #3 = 90º.

### Data processing and statistical analysis

Data processing was performed in MATLAB® (TheMathworks Inc., Natick, MA, USA). A 23 Hz gaussian line broadening was applied prior to Fourier transformation. Each real spectrum was then corrected for phase, baseline and baseline offset. The first six spectra for each frequency range were summed. The areas under the spectral peaks of interest were calculated by integration and normalized to the [2-^13^C]DHAc integral to minimize variations due to [2-^13^C]DHAc polarization, concentration, volume of infusion, tracer delivery, and coil loading and placement [11]. Average data are shown as mean ± standard deviation for the three animals.

**Fig. 4:**
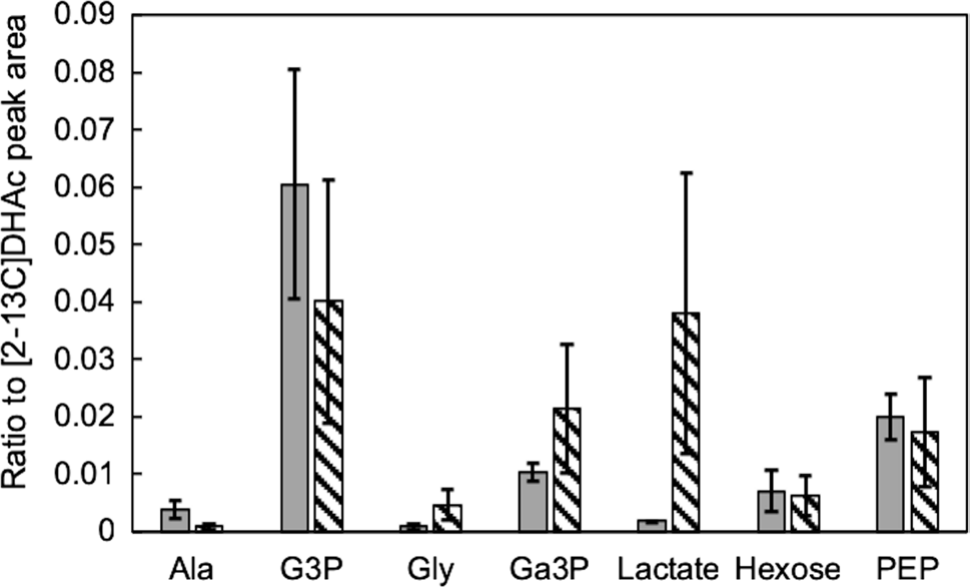
Ratio of metabolite peak areas to that of dihydroxyacetone (DHAc) 20 s after the injection of a bolus of the hyperpolarized ^13^C-labeled substrate. The mean ratio is shown in grey with the standard deviation indicated by an error bar. The diagonal striped bars indicate the calculated ratios at 20 s mark after the start of DHAc infusion based on the analysis described by Kirpich et al. [23]

Estimated metabolite-to-substrate ratios were calculated at *t* = 20 s based on the ex vivo results and model of Kirpich et al. [23]. The rate constants and relaxation time constants included in the model were not adjusted for field strength or in vivo conditions. Details on the simulations are given as supporting information in S1. Briefly, a gamma function (Eq. 11 in reference [23]) was used, with *α* = 1 and *β* = 1, and the time courses of the 14 metabolites included in the model were simulated with an ordinary differential equation solver at *t* = 20 s. This was then repeated with *α* = 2/*β* = 2 and *α* = 3/*β* = 3 to generate the data in Fig. S1, showing that for many metabolite ratios the input function does not perturb the result greatly. The simulation is again repeated with *α* = 1/*β* = 1 and 11 values of *k* drawn randomly from a normal distribution with the mean and standard deviation values from Kirpich et al. [23] This is repeated 1000 times with any negative values of rate constants discarded and replaced. The mean (bar value) and standard deviation (error bar) from these 1000 simulations are plotted as the modelled data in Fig. 4.

## Results

The RF and magnetic field gradient waveforms, and RF excitation spectral–spatial profiles of the resulting three purpose-designed SPSP RF pulses are shown in Fig. 2a–f. ‘Pulse #1’ (10.44 ms) excited the [2-^13^C]DHAc resonance at 214 ppm; ‘pulse #2’ (10.18 ms) the PEP resonance at 150.5 ppm; and ‘pulse #3’ (2.98 ms) excited other metabolites of interest at around 72 ppm and 50 ppm—namely C5-β-glucose-6-phosphate (G6P-C5), C2-3-phosphoglycerate (3PG-C2), C2-β-glucose (Glc-C2), C2-β-glucose-6-phospate (G6P-C2), C2-glyceraldehyde 3-phosphate (Ga3P-C2), C2-glycerol (Gly-C2), C2-glycerol-3-phosphate (G3P-C2), C2-lactate (Lac-C2) and C2-alanine (Ala-C2).

Representative ^1^H-decoupled ^13^C-MR spectra acquired sequentially in vivo 20 s after tail vein injection of HP [2-^13^C]DHAc across the three separate spectral regions are shown in Fig. 3b–d. Positioning of the receiver coil is shown in Fig. 3e and f and the full spectral ranges acquired for each spectrum are displayed in Fig. S2. ^1^H-decoupling collapsed the G3P-C2 and Ga3P-C2 resonance doublets into singlets and also improved the resolution of smaller signals, such as glucose, G6P, lactate-C2 and alanine-C2. The resonances observed following pulse #3 (Fig. 3d) were assigned by reference to a previous study performed in perfused livers in a glycogenolytic state [10], where the same resonances were observed after the infusion of hyperpolarized [2-^13^C]DHAc.

The integrals of the metabolite peaks observed in Fig. 3b–d were calculated. The integrals of the C2-β-glucose (Glc-C2), C2-β-glucose-6-phosphate (G6P-C2), C5-β-glucose-6-phosphate (G6P-C5) and C2-3-phosphoglycerate (3PG-C2) resonances, which were not well resolved, were added together to give a collective value for the hexoses. Table 1 summarizes the in vivo metabolite-to-substrate ratios obtained in the three mice (the spectra from which these ratios were obtained are displayed in Fig. S3).

**Table 1:** Metabolite-to-[2-^13^C]DHAc signal intensity ratios (*n* = 3)

	Ala	G3P	Gly	Ga3P	Lac	Hex	PEP
Metabolite ratio ± SD	0.0038 ± 0.0015	0.0605 ± 0.0200	0.0009 ± 0.0005	0.0102 ± 0.0016	0.0018 ± 0.0001	0.0070 ± 0.0035	0.0199 ± 0.0040

*Ala* alanine, *G3P* glycerol-3-phosphate, *Gly* glycerol, *Ga3P* glyceraldehyde 3-phosphate, *Lac* lactate, *Hex* hexoses, *PEP* phosphoenolpyruvate

The metabolite-to-substrate ratios measured in vivo at 20 s after injection of hyperpolarized [2-^13^C]DHAc were compared with those estimated at the same time point from the ex vivo results obtained in perfused livers (Fig. 4) [23]. The estimated ratios are relatively insensitive to the shape of the input function, except for lactate-C2 (see Fig. S1). In some cases, the ratios were similar, including the ratio for the combined hexose resonances (mostly glucose and G6P). However, the Ga3P signal was slightly greater than half the expected amplitude estimated from the ex vivo data while the lactate and alanine ratios were very different between the two studies.

## Discussion

We have demonstrated that HP [2-^13^C]DHAc enables simultaneous detection of intermediates in GNG, glycolysis and glycerol synthesis in vivo with a spectral resolution and sensitivity similar to that reported in perfused mouse liver [10]. To achieve this, excitation was performed using a train of optimized SPSP RF pulses and ^13^C acquisition with broadband ^1^H-decoupling. A SPSP RF pulse had been used previously at 3 T to excite the broad spectrum of interest (> 144 ppm), while minimizing excitation of DHAc and preserving magnetization of the metabolites beyond DHAP [18]. With the higher field used here (7 T), and thus greater spectral dispersion of the metabolite resonances, it was possible to use three sequential SPSP RF pulses to excite three distinct spectral regions. The 10 mm surface coil placed directly over the liver and the SPSP RF pulses ensured that the excited and observed volumes resided within the liver and its associated vasculature.

A potential concern related to the application of ^1^H decoupling during in vivo ^13^C MR acquisitions is local tissue heating. However, specific absorption rates (SAR) can be taken into account during the RF pulse and coil design. Decoupling schemes have been implemented previously in human studies [24]. Here, conventional broadband ^1^H-decoupling was applied exclusively during acquisition and nuclear Overhauser effect (NOE) enhancement from ^1^H to ^13^C was therefore avoided.

Following injection of HP [2-^13^C]DHAc, the metabolic products observed with highest SNR were G3P, Ga3P, PEP and alanine. As in the previous studies performed in vivo at 3 T [11] and in the perfused liver at 9.4 T [10], it was not possible to resolve the DHAP resonance from the large substrate signal.

A single HP [2-^13^C]DHAc injection was given to each animal and, as has been shown previously in rats using a glucometer blood reading [11], we assume that this does not perturb circulating glucose levels. The ratio of labeled 3-carbon metabolites (G3P, Ga3P, PEP, Lac, Ala, Gly) to hexoses was 14.9 ± 3.2, which indicates higher glycolytic than gluconeogenic activity. This agrees with a study where hepatocytes harvested from fed rats incubated with DHAc showed that ~ 90% of the total DHAc was converted into the downstream metabolites of glycolysis, and only ~ 10% was converted into glucose [25].

The acquisition method used here was different from that used in the previous ex vivo studies: (1) the field strength was higher—which may affect some of the metabolite T_1_s and T_2_s—and (2) in vivo we used a bolus of DHAc rather than a 4 mM infusion over 90 s [10]. This difference in the delivery of the substrate was necessary to optimize the SNR in the in vivo (7 T) experiment. The spectra of fed mouse liver in vivo observed here are remarkably similar to those observed ex vivo despite the differences in the experimental protocols. Although the datasets are significantly different for each metabolite ratio measured and simulated, the similarities in relative metabolite amounts are striking given the expected errors in the acquired data and the modelling. Variations between the in vivo and ex vivo datasets are most likely due to differences in the bolus injection of [2-^13^C]DHAc, which could lead to saturation of transporters or enzymes in vivo, and changes in the relative flux through the pathways. For example, the Ga3P-to-DHAc ratio measured in vivo was lower than in perfused liver, which will be highly dependent on the relative activities of triose phosphate isomerase and G3P dehydrogenase (which is dependent on NADH and therefore redox status). It is likely that the flux from DHAc to Ga3P is lower in the in vivo experiment due to differing experimental conditions. In contrast, the alanine-to-lactate ratio of 2.2 ± 0.9 was substantially higher in the in vivo experiments as compared to the previous studies in perfused liver, where it was less than 1 [26, 27], suggesting greater alanine transaminase activity in vivo or reduced lactate dehydrogenase activity ex vivo caused by lower NADH concentration. It should be noted that the implementation of the model—here in these simulations—leads lactate and alanine amounts to be highly dependent on the input function used, increasing their variability at a simulated 20 s time point.

Under physiological conditions, GNG and glycolysis are balanced according to the metabolic state of the liver such that one dominates over the other according to need. Despite this, imaging with a surfeit of DHAc (the concentration of DHAc in normal liver is 13 µmol/kg in rats [28]) shows the capacity of at least parts of these metabolic processes to occur simultaneously.

The limited SNR restricted the present study to a static snapshot of liver metabolism and it was, therefore, not possible to use kinetic models to analyze the data, unlike in the perfused liver study [23]. Future efforts should focus on higher hyperpolarization levels, a faster and automated injection method [29], and larger animal models to allow dynamic and spatial measurements.

To conclude, we have demonstrated that hyperpolarized [2-^13^C]DHAc can be used to probe triglyceride-precursor synthesis and glucose metabolism in real time in the liver in vivo using a combination of ^1^H-decoupling and SPSP RF pulses. This protocol should further enable changes in glucose metabolism to be investigated non-invasively in different metabolic states, including those caused by cancer and NAFLD. For example, Changani et al*.* reported that patients with cirrhotic liver showed reduced GNG from alanine using ^31^P-MR spectroscopy [30]. Similarly, reduced conversion of HP DHAc into glucose should indicate hepatocyte cell-loss or function-loss and also localize the region affected for biopsy. If so, HP DHAc may prove a sensitive test for pre-cancerous and cancer lesions.

## Electronic supplementary material

Below is the link to the electronic supplementary material.

Supplementary file1 (PDF 1231 kb)
