# Supplementary material for: Probing hepatic metabolism of [2-13C]dihydroxyacetone in vivo with 1H-decoupled hyperpolarized 13C-MR
Source: MAGMA. 2020 Sep 10;34(1):49–56. doi: 10.1007/s10334-020-00884-y (PMC7910257; doi:10.1007/s10334-020-00884-y)

Supporting Information

Probing hepatic metabolism of [2-^{13}C]dihydroxyacetone in vivo with ^{1}H-decoupled hyperpolarized ^{13}C-MR

Irene Marco-Rius[1*x], Alan J. Wright[1*], De-en Hu[1], Dragana Savic[2,4], Jack J. Miller[2,3,4], Kerstin N. Timm[2], Damian Tyler[2,4], Kevin M. Brindle[1], and Arnaud Comment[1,5]

[1] Cancer Research UK Cambridge Institute, University of Cambridge, UK

[2] Department of Physiology, Anatomy and Genetics, University of Oxford, UK

[3] Clarendon Laboratory, Department of Physics, University of Oxford, UK

[4] Oxford Centre for Clinical Magnetic Resonance Research, Radcliffe Division of Medicine, University of Oxford, UK

[5] General Electric Healthcare, Chalfont St Giles, UK

These authors contributed equally to this work.

[4] Corresponding author: Irene Marco-Rius, imarco@ibecbarcelona.eu

[x] Current address: Institute for Bioengineering of Catalonia, Barcelona, Spain

Fig. S1 (A) Calculated metabolite-to-substrate ratios 20 s after the start of substrate infusion using three different input functions (B). The simulations show that the metabolite ratios, with the exception of that of lactate, are largely insensitive to large variations in the shape of the input functions. Additional information on simulation parameters and model can be found below (S1).

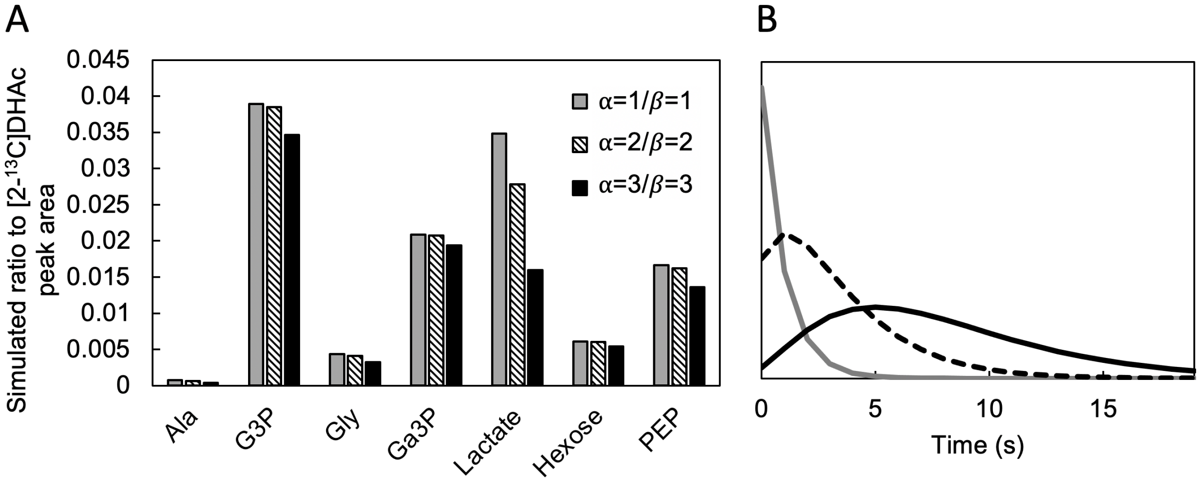

S1. Additional information on simulation model and parameters used in Fig. S1.

Simulations were based on the model and kinetic constants for fed mice from Kirpich *et al.*[1], which used the 14 metabolites and corresponding T_1 relaxation time constants shown in the table below:

Metabolite	T_1 (seconds)
DHAc	65
DHAP	36
Ga3P	3.1
2G6P	1.9
2Glc	2.8
5G6P	2.6
5Glc	3.1
3PG	3.9
PEP	15
Pyr	16
Lac	6.8
Ala	5.5
G3P	2.4
Gly	3.1

Moreover, the following set of differential equations were used (see Fig. 1 of Kirpich et al.[1]):

$d[DHAc]/dt=-R_{DHA}*[DHAc]-k_1*[DHAc]+F_{input}$

$d[DHAP]/dt=-R_{DHAP}*[DHAP]+k_1*[DHAc]-k_2*[DHAP]-k_4*[DHAP]-k_{10}*[DHAP]$

$d[Ga3P]/dt=-R_{Ga3P}*[Ga3P]+k_4*[DHAP]-k_5*[Ga3P]-k_2*[DHAP]$

$dy[2G6P]/dt=-R_{2G6P}*[2G6P]+k_2*[DHAP]-k_3*[2G6P];$

$dy[2Glc]/dt=-R_{2Glc}*[2Glc]+k_3*[2G6P];$

$dy[5G6P]/dt=-R_{5G6P}*[5G6P]+k_2*[Ga3P]-k_3*[5G6P]$

$dy[5Glc]/dt=-R_{5Glc}*[5Glc]+k_3*[5G6P]$

$dy[3PG]/dt=-R_{3PG}*[3PG]+k_5*[Ga3P]-k_6*[3PG]$

$dy[PEP]/dt=-R_{PEP}*[PEP]+k_6*[3PG]-k_7*[PEP]$

$dy[Pyr]/dt=-R_{pyr}*[Pyr]+k_7*[PEP]-k_8*[Pyr]-k_9*[Pyr]$

$dy[Lac]/dt=-R_{Lac}*[Lac]+k_8*[Pyr]$

$dy[Ala]/dt=-R_{Ala}*[Ala]+k_9*[Pyr]$

$dy[G3P]/dt=-R_{G3P}*[G3P]+k_{10}*[DHAP]-k_{11}*[G3P]$

$dy[Gly]/dt=-R_{Gly}*[Gly]+k_{11}*[G3P]$

where

2Glc/2G6P refers to the carbon-13 labelling at position 2 of glucose and glucose-6-phosphate, respectively.

$R_{metabolite}=1/T_{1,metabolite}$ and

F_{input} is a gamma function ($F=t^{\alpha-1}e^{-t/\beta}$).

$k_1=0.03\pm0.015$; $k_2=0.031\pm0.008$; $k_3=0.355\pm0.035$; $k_4=0.34\pm0.005$; $k_5=0.349\pm0.03$; $k_6=3.48\pm0.04$; $k_7=0.39\pm0.015$; $k_8=0.32\pm0.0001$; $k_9=0.008\pm0.0001$; $k_{10}=0.39\pm0.0001$; $k_{11}=0.032\pm0.004$.

A MATLAB ordinary differential equation solver was used to simulate the time courses of this set of equations. An initial simulation used the mean values of the kinetic constants and $\alpha=1/\beta=1$ to generate the metabolite ratios at 20 s of Ala, G3P, Gly, Ga3P, Lac and PEP over DHAc and the combined hexose (2G6P+2Glc+5G6P+5Glc+3PG) over DHAc. This was then repeated with $\alpha=2/\beta=2$ and $\alpha=3/\beta=3$ to generate the data in Fig. S1, showing that for many

metabolite ratios the input function does not perturb the result greatly. The simulation was again repeated with α=1/β=1 and values for k_1 to k_{11} drawn randomly from a normal distribution with the mean and standard deviation values from Kirpich et al.[1] as given above. This was repeated 1000 times with any negative values of rate constants discarded and replaced. The mean (bar value) and standard deviation (error bar) from these 1000 simulations are plotted as the modelled data in Fig. 4.

Note the small differences in a single simulation with mean rate constants (plotted in Fig. S1) and the mean values from 1000 simulations (plotted in Fig. 4). A student t-test of the 1000 simulations suggests that their mean values for final metabolite ratios are statistically significant from the mean in vivo metabolite ratio in all instances.

Reference

(1) Kirpich, A.; Ragavan, M.; Bankson, J. A.; McIntyre, L. M.; Merritt, M. E. Kinetic Analysis of Hepatic Metabolism Using Hyperpolarized Dihydroxyacetone. *J. Chem. Inf. Model.* **2019**, *59* (1), 605–614. https://doi.org/10.1021/acs.jcim.8b00745

Fig. S2 Magnitude spectra of the three mouse livers showing the resonances of the observed metabolites. The frequency-excitation bands of the spectral-spatial pulses are indicated with green frames. The three panels show the frequency ranges of pulses #1 (top), #2 (middle) and #3 (bottom). The top panel shows the signal obtained from the injected [2-^{13}C]dihydroxyacetone (DHAc), the middle panel signal from phosphoenolpyruvate (PEP). Multiple frequency bands are shown for pulse #3 to indicate that alanine-C2 (Ala) is excited as well as the glycerol-3-phosphate-C2 (G3P) region and the second carbon of dihydroxy-acetone hydrate (DHAc hydrate).

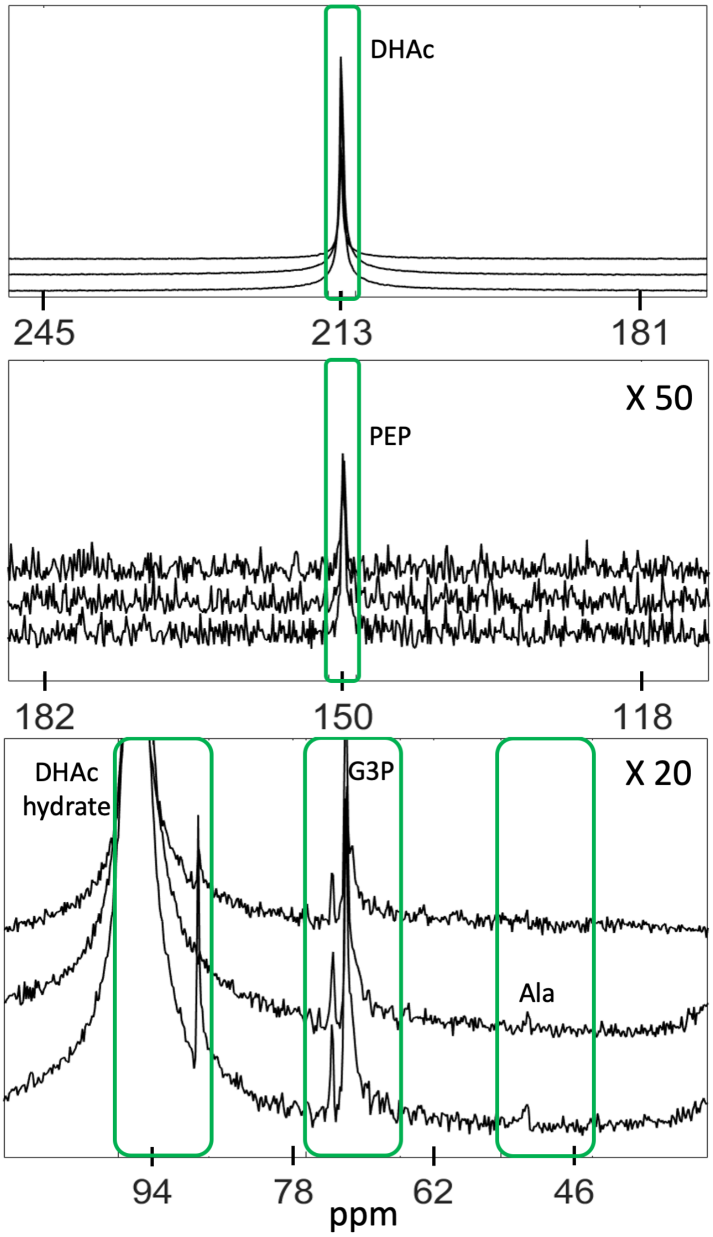

Fig. S3 Summed spectra acquired using pulse #3 from the three mouse livers.

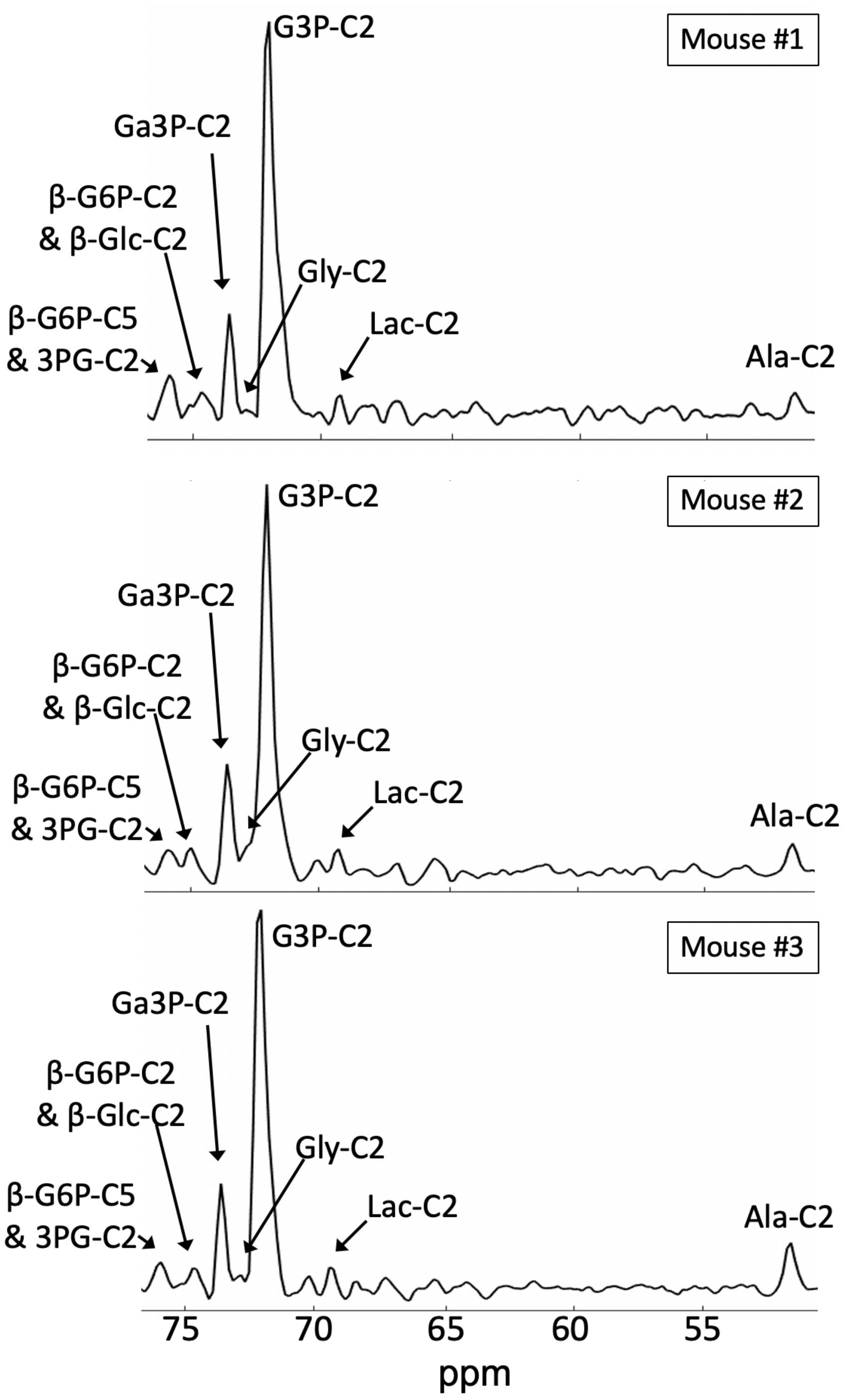

Supplement: Supplementary file 1 — Supplementary file1 (PDF 1231 kb) [file 10334_2020_884_MOESM1_ESM.pdf]
